# Formulation of *Mentha piperita*-Based Nanobiopesticides and Assessment of the Pesticidal and Antimicrobial Potential

**DOI:** 10.3390/life14010144

**Published:** 2024-01-19

**Authors:** Nazish Jahan, Nida Hussain, Syeeda Iram Touqeer, Khalil-Ur-Rahman, Huma Shamshad, Naseem Abbas

**Affiliations:** 1 Department of Chemistry, University of Agriculture, Faisalabad 38000, Pakistan; nazishjahan@uaf.edu.pk (N.J.); nidahussain116@gmail.com (N.H.); iram_touqeer@yahoo.com (S.I.T.); dogarhuma@gmail.com (H.S.); 2 Department of Biochemistry, Riphah International University, Faisalabad 38000, Pakistan; khaliluaf@yahoo.com; 3 Department of Mechanical Engineering, Sejong University, Seoul 05006, Republic of Korea

**Keywords:** *Mentha piperita*, nanobiopesticides, *Tribolium castaneum*, *Sitophilus oryzae*

## Abstract

The excessive use of synthetic pesticides has detrimental impacts on humans, non-target organisms, and the environment. Insect pest management strategies are shifting toward biopesticides, which can provide a feasible and environmentally friendly green solution to the pest problem. The key objective of the present research work was the preparation of *Mentha piperita*-based nanobiopesticides with enhanced stability, solubility, and pesticidal potential. Nanobiopesticides based on the *Mentha piperita* extract were prepared using the antisolvent precipitation method. The central composite design of response surface methodology (RSM) was utilized to optimize different process parameters, e.g., the amounts of the stabilizer and plant extract. The nanosuspension of *Mentha piperita* prepared with the stabilizer SLS showed a particle size of 259 nm and a polydispersity index of 0.61. The formulated biopesticides in the form of nanosuspensions showed good antibacterial activities as compared to the *Mentha piperita* extract against two phytopathogenic bacterial strains, *Clavibacter michiganensis* and *Pseudomonas syringae*. The *M. piperita* nanosuspension had higher antifungal efficacy against *A. niger and F. oxysporum* than the *Mentha piperita* extract. The *M. piperita* extract and its nanosuspensions were tested for pesticidal activity against the stored-grain insects *Tribolium castaneum* and *Sitophilus oryzae. Mentha piperita*-based nanobiopesticides demonstrated significantly high (*p*  <  0.05) average mortality of 84.4% and 77.7% against *Tribolium castaneum* and *Sitophilus oryzae*, respectively. *Mentha piperita*-based nanobiopesticides showed enhanced pesticidal potential and could be used as a good alternative to synthetic chemical pesticides.

## 1. Introduction

Pesticides are chemical or biological substances used to kill insects, plant pathogens, and weeds in the agricultural field. Approximately 5.2 billion pounds of pesticides are used worldwide every year [1]. However, a large proportion of pesticides are wasted during their application through runoff, leaching, seepage, microbial degradation, and photodegradation. Only a small volume (<1%) of the applied pesticide becomes available to the target pest [2]. Synthetic pesticides such as organochlorines, pyrethroids, organophosphate, and carbamates have been used in the past for crop protection because of their effectiveness, broad spectrum of action, low cost, easy usage, and speed of action [3]. Their indiscriminate and uncontrolled use causes ecotoxicity because they are persistent compounds, and their non-degradable residual product causes soil and groundwater pollution. These chemicals adversely affect non-target organisms and cause resistance in the target pest [4,5]. These pesticides accumulate in humans and cause acute skin and eye irritation, dizziness, nausea and headaches, and chronic effects like carcinogenicity, teratogenicity, spermatotoxicity, genotoxicity, hormonal imbalance, diabetes, and asthma [6].

Biopesticides are derived from natural materials, like plants, animals, microorganisms, and minerals. They are considered ecofriendly alternatives to conventional pesticides because they are less poisonous, less persistent, biodegradable, highly specific to their target pests, and needed in small amounts [7]. Depending on the nature of the active ingredient and mode of action, biopesticides are generally classified into three classes: microbial pesticides, biochemical pesticides, and plant-incorporated protectants [8]. The pesticidal potential of *Cymbopogon citratus, Mentha spicata, Origanum vulgare, Thymus serpyllum, Tanacetum vulgare, Pimpinella anisum,* and *Jatropha curcas* has been reported previously [8,9,10,11,12,13]. The rapid development and utilization of biopesticides are hindered due to costly production methods, pest control selectivity, UV degradation, inadequate storage ability, slow speed of action, susceptibility to adverse environmental conditions, and irregular practical use in the field. The usage of biopesticides is >1% as compared to total pesticide usage in the market [14].

“Nanobiopesticides” are superior to “mere biopesticides”, as they show enhanced bioavailability, biocompatibility, biodegradability, and no premature deterioration. These factors increase the stability of pesticides, along with enabling the controlled and sustainable release of active ingredients in response to certain environmental triggers. Nanopesticides are required in small dosages and are ecofriendly. The delivery systems of nanopesticides have the potential to activate and monitor the release profile at a distance [15,16,17]. Among various nanotechnological methods, nanosuspension technology has developed as a promising approach to enhancing the oral bioavailability, dissolution, and solubility of insoluble drugs [18]. Nanosuspensions are surfactant-stabilized submicron colloidal dispersions of nanoparticles, generally prepared by a top-down approach or bottom-up approach. Many studies have proved that nanoemulsions of sweet orange, grapefruit, garlic, camelthorn, eucalyptus, citronella, and neem oils can be used as botanical pesticides [19,20,21]. Similarly, the insecticidal potential of *Blumea balsamifera*, *Pedalium murex*, *Lobelia leschenaultiana*, *Suaeda maritima*, *Sargassum muticum*, and *Mussaenda glabrata* based nanoparticles has been reported in previous literature [22,23].

*Mentha piperita* is an important aromatic and medicinal plant of the Lamiaceae family. Essential oils extracted from different parts of plants are widely used in the cosmetic, food, health, and pharmaceutical industries [24]. The main components of EOs include menthone, limonene, methyl acetate, menthol, isomenthol, terpinene, menthofuran, cineol, tannin, pinene, etc. Mint extract is known for its antiseptic, antibacterial, antifungal, antispasmodic, stimulant, and carminative effects [25]. Its herbal tea is useful for the treatment of gastrointestinal diseases, musculoskeletal pains, menstrual and stomach cramps, indigestion, vomiting, flatulence, and parasitoids. The repellent and insecticidal potential of *M. piperita* has also been studied using mosquitos, houseflies, and stored-product insects [26].

Some researchers prepared nanoparticles (Ag, Zn) of *M. piperita* to improve its insecticidal and antimicrobial potential [27]. However, no research has been conducted on the nanoformulation of the *Mentha piperita* extract. Therefore, the key purpose of this study was the preparation and characterization of a *Mentha piperita* nanosuspension and the evaluation of its enhanced pesticidal potential.

## 2. Materials and Methods

### 2.1. Preparation of Mentha piperita Extract

The *Mentha piperita* leaves were collected from the botanical garden of Agriculture University Faisalabad. The leaves were green on top, pale green on the bottom, and glabrous. *Mentha piperita* leaves were cultured by the ancient Egyptians and recognized in the Icelandic pharmacopoeia of the 13th century. It is widely grown in temperate areas of the world, particularly in North Africa, Europe, and North America, but is nowadays cultivated throughout all regions of the world. The Mentha leaves were washed, dried in the shade, and ground into powder form. A Soxhlet apparatus was used for plant extraction using ethanol as a solvent. For this purpose, 30 g of plant material was placed in the Soxhlet apparatus, 300 mL of ethanol (solvent) was added to a round-bottom flask, and the extraction procedure proceeded for 12 h. The prepared plant extract was concentrated on a hot plate at 40 °C for 1 h and stored in the refrigerator (4 °C) for further study.

### 2.2. Nanosuspension Formulation and Characterization

Nanosuspensions of *Mentha piperita* extract were prepared using the solvent/antisolvent precipitation method [28]. Briefly, *Mentha piperita* extract (0.25 g) was dissolved in 10 mL of ethanol (organic phase) and added dropwise (1 drop/s) through a syringe into an aqueous medium (100 mL) containing a stabilizer, such as Tween-80, polyethylene glycol, polyvinyl alcohol, sodium lauryl sulfate, polyvinyl pyrrolidone, or Hydroxypropyl methyl cellulose, and the mixture was continuously stirred for 5 h at 3000 rpm [29]. A Malvern zeta sizer was used for measuring the particle size (nm) and polydispersity index (PDI) values of the formulated nanosuspensions through the dynamic light scattering (DLS) method. The stabilizer that produced the nanosuspension with the smallest particle size and PDI values was selected for further studies.

### 2.3. Optimization of Nanosuspension

After choosing one suitable stabilizer for the *Mentha piperita* extract, the central composite design of response surface methodology (RSM) was used for the optimization of the independent parameters (amounts of stabilizer and plant extract) [30]. Various preliminary trials were applied to select suitable ranges, which were 0.20–1 g and 0.20–1.35% for the plant extract and stabilizer concentrations, respectively. The response or dependent parameter for the nanosuspension was particle size. The design of the experiment was used to optimize the selected parameters. Based on the number of factors and their levels, the central composite design (CCD) was used to investigate the effects of various parameters on the physical properties of the prepared nanosuspensions. The design consisted of a total of 13 experiments, and the data obtained via these experiments were analyzed using Design-Expert software (Minneapolis, MN, USA). The optimized nanosuspensions were placed at two different temperatures, at 25–30 °C (room temperature) and at 4 °C (refrigerator), to evaluate the physical stability of the nanosuspensions for a three-month duration [31]. After storage, the PDI and zeta size were determined.

### 2.4. Determination of Pesticidal Activity

The pesticidal potential of the *Mentha piperita* extract and the optimized nanosuspensions at different concentrations (0.60, 1, and 1.17%) and time intervals were evaluated against *Tribolium castaneum* (red flour beetle) and *Sitophilus oryzae* (rice weevils) in Petri plates. Twenty *Sitophilus oryzae* and *Tribolium castaneum* insects were placed in each Petri dish and appropriately covered after the administration of the sample to determine the mortality rate of both pests after 24, 48, and 72 h [32]. The following equation was used to determine the mortality rate (%):

Ma(%) = (Mp − Mc) × 100

where Ma represents the actual mortality rate (%), Mp represents the observed mortality rate of pest treatments (%), and Mc represents the mortality rate of the control (%).

### 2.5. Determination of Antimicrobial Activity

The antimicrobial potential of *Mentha piperita*-based nanobiopesticides was examined against two bacterial and fungal strains using the agar well diffusion method.

#### 2.5.1. Antibacterial Activity

Two bacterial strains, *Clavibacter michiganensis* (Gram-positive) and *Pseudomonas syringae* (Gram-negative), were selected for antibacterial screening. In a conical flask, 500 mL of distilled water was added to 11.5 g of nutrient agar, stirred well, and covered with aluminum foil to make the nutrient agar solution. After mixing the solution, it was placed in a 121 °C oven for 15 min. After that, in 50 mL of distilled water, 0.25 g of yeast, 0.25 g of NaCl, and 0.7 g of tryptophan were mixed and autoclaved to make a broth solution for the culture of bacterial strains. When the solution was ready, 150 µL of each prepared bacterial culture was added to the flask containing the agar medium in a laminar flow, and then this medium was poured into a sterilized Petri plate and allowed to cool. After the agar medium had solidified, a well of 4 to 5 mm was made on a Petri plate using a wire loop. Then, 50 µL samples of various concentrations (1.5, 2, 3%) of the formulated nanobiopesticides and *Mentha piperita* extract were added to these wells, and the Petri plates were kept overnight in an oven at 37 °C. As a positive control, the antibiotic ciprofloxacin was utilized. After 24 h, the zone of inhibition developed in the Petri plates was quantified using a zone reader in mm [33].

#### 2.5.2. Antifungal Activity

The antifungal activity was evaluated against *Asperlligus niger* and *Fusarium oxysporum* using the agar well diffusion method. The culture medium of both fungal strains was collected from the Fungal Molecular Biology Laboratory Culture Collection (FMB-CC), University of Agriculture Faisalabad, Pakistan. To begin, the agar medium (PDA) was made by mixing 500 mL of distilled water and 21 g of potato dextrose agar in a conical flask. Then, this solution was autoclaved for 20 min at 120 °C. After cooling, PDA media were poured into sterilized Petri dishes, and 2 µL of each fungal strain was added to these Petri plates. Wells were created with a borer, and 80 µL samples of varying concentrations (1.5%, 2%, and 3%) of plant extract and nanobiopesticides were added into these wells. A synthetic antifungal tablet, voriconazole, was used as a control. The Petri dishes were placed at 37 °C for 72 h. After incubation, a Zone reader was used to measure inhibition zones in mm [34].

### 2.6. Statistical Analysis

All experiments were performed in triplicate (*n* = 3) and are presented as the mean ± standard error. Data were investigated by using ANOVA *p* < 0.05, followed by Tukey’s multiple-comparison test.

## 3. Results and Discussion

### 3.1. Screening of Stabilizers for Nanosuspension Formulation

**Figure 1:**
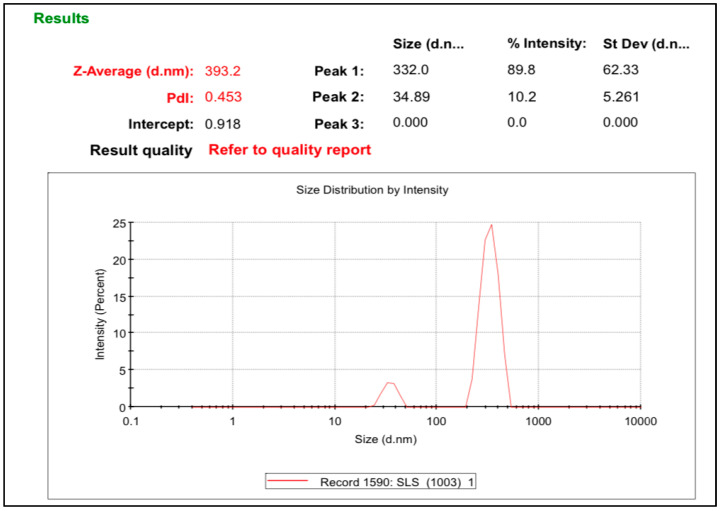
Particle size of *Mentha piperita* nanosuspension prepared with SLS.

*Mentha piperita*-based nanobiopesticides in the form of nanosuspensions were formulated. The mean particle size (Z-average; nm) and polydispersity index (PDI) of the formulated nanosuspensions were evaluated using the dynamic light scattering technique. Pharmaceutically safe stabilizers, e.g., Tween-80, HPMC, PVP, SLS, PVA, and PEG, were used to make different nanosuspensions of the *Mentha piperita* extract. The nanosuspensions prepared with HPMC, PVP, PVA, and PEG became unstable at room temperature, while nanosuspensions containing Tween-80 or SLS remained stable. The nanosuspension prepared with SLS had a reduced particle size of 393 nm. The PDI value of 0.4 with SLS was also appropriate (Figure 1); hence, it was chosen for the next activities. The SLS stabilizer adheres to the surface of the particles, causing electrostatic repulsion and preventing the accumulation of nanoparticles [35]. The stabilizer plays a significant role in maintaining the physical stability of nanosuspensions. A small particle size with a high surface area may increase the interfacial tension and free energy (ΔG) of the system, which is responsible for the crystal growth or particle aggregation. Surfactants prevent particle agglomeration by decreasing the free energy and interfacial tension, thus preventing Ostwald ripening [28,29]. They cause electrostatic repulsion or steric hindrance to stabilize the smaller particles, so the use of a stabilizer is essential for the formulation of nanobiopesticides.

### 3.2. Optimization of Process Parameters of Nanosuspension by RSM

**Figure 2:**
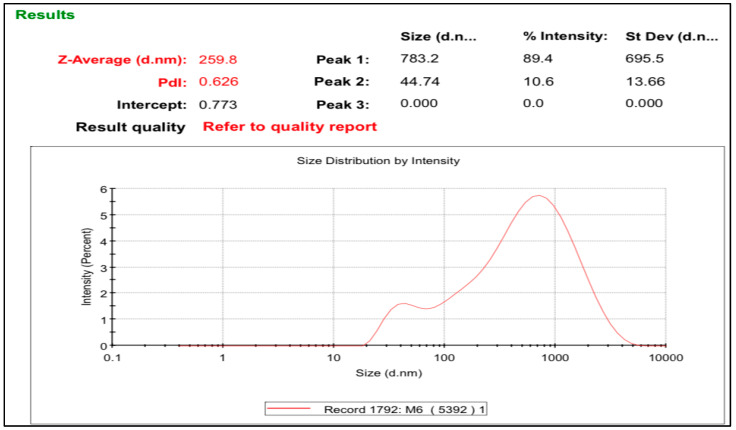
PDI and particle size values of optimized *Mentha piperita* nanosuspensions.

It is necessary to optimize the formulation parameters to develop an efficient and pharmaceutically stable nanosuspension with the desired morphology and particle size. The optimization of each factor is expensive and time-consuming, and it does not reveal the influence of individual independent variables on different dependent variables. Secondly, a factorial design requires various trials that fail to give useful information (interactive effect), thus complicating the matter. To address these issues, the central composite design of RSM can be effectively employed for optimization studies. The response or dependent parameter for the nanosuspension was particle size. The Design-Expert software (version 7.1, Stat-Ease, Inc., Minneapolis, MN, USA) generated polynomial equations and response variables. The RSM response optimization function was used to determine the optimal values of the independent variables. After selecting one appropriate stabilizer, i.e., SLS (sodium lauryl sulfate), for the formulation of the *Mentha piperita* nanosuspension, the central composite design of response surface methodology was utilized to optimize independent factors, such as the stabilizer concentration and plant extract amount. Several preliminary trials were conducted to select the appropriate ranges, which were 0.20–1 g for the plant extract and 0.20–1.35% for the stabilizer content. All nanosuspensions were prepared with the same antisolvent (water)-to-solvent (ethanol) ratio. The optimization was based upon the minimum particle size and lower PDI value. Based on the central composite design (CCD), a set of 13 experiments were designed with 7.1 Design-Expert software. The optimized nanosuspension with a nanoparticle size of 259.8 nm and a PDI of 0.6 was produced with 1% *Mentha piperita* extract and 0.20% stabilizer concentration, as shown in Figure 2. The largest particle size of the *Mentha piperita* nanosuspension was 431 nm, which was observed at 0.20% *Mentha piperita* extract and a 0.20% SLS concentration. The observed ranges of the PDI and particle size were 0.378–0.626 and 259.8–431 nm, respectively. The initial step in the optimization analysis was the selection of a suitable model (Table 1).

The RSM will develop several models (linear, two-factor interaction (2FI), linear, cubic, and quadratic polynomial) that fit the response and offer the best model. A highly significant model has a *p*-value of less than 0.0001, while *p* < 0.05 shows a significant model. The coefficient of determination is often used to determine the model’s accuracy. A 100% or close-to-100% value of R^2^ shows that the predicted and actual responses are closely related. On the basis of the values of responses obtained, the software predicted a quadratic model for the particle size of the nanosuspension. The impact of the independent parameters on the response variable was evaluated by creating a regression equation for the response. The particle size of the nanosuspension was expressed as a function of the independent factors using a second-order polynomial equation.

### 3.3. Polynomial Equation in Terms of Coded Factors

For optimization, a 3^2^ factorial design generated a mathematical model equation that involves independent factors and their interactions for different measured responses:

(1) $$y=b_{0}+b_{1}X_{1}+b_{2}X_{2\;}+b_{12}X_{1}X_{2}+b_{11}\;X_{1}^{2}+b_{22}X_{2}^{2}$$

The dependent variable is Y, the arithmetic mean response of 13 runs is b_0_, and the estimated coefficient for the *X*_1_ factor is represented by *b*_1_. The main effects (*X*_1_ and *X*_2_), interaction terms (*X*_1_*X*_2_), and polynomial terms (*X*_1_^2^ and *X*_2_^2^) represent the average results of varying one independent factor at a time from its lowest to its highest value, those of varying two factors simultaneously, and nonlinearity, respectively.

Particle size(nm) for *Mentha piperita* Nanosuspension = +278.86 − 42.19A + 14.07B + 41.60AB + 47.53A^2^ + 23.35B^2^ (2)

A positive sign before the stabilizer value indicates a synergistic effect, while a negative sign before the plant extract shows an antagonistic influence, as reported by Singare, et al. [35]. The analysis of variance (ANOVA) for the particle size of the nanosuspension is given in Table 2.

**Figure 3:**
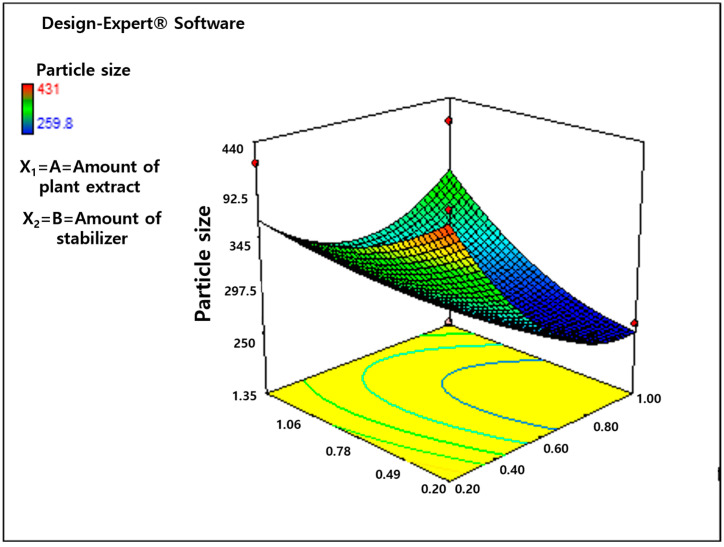
Three-dimensional graph representing combined effect of stabilizer concentration.

The F-value (3.97) and *p*-value (0.0499) show the significance of the model at a confidence level of 95%. In this situation, a *p*-value below 0.05 indicates that the specific model terms were statistically significant. The fitness and significance of the quadratic model were examined using an analysis of variance [36]. The lack-of-fit *p*-value of 0.0510 (*p* < 0.05) and the F-value of 6.51 showed no significant lack of fit relative to the pure error. The model is good if the R^2^ value is near unity, as it shows a fair agreement between actual and predicted yields. The observed R^2^ value was 0.7395, which means that 73% of the change in particle size was due to the given experimental independent variables. A CV of 14 was observed for the particle size of the Mentha piperita nanosuspension. Regression functions are graphically represented by a two-dimensional line graph and a three-dimensional response surface graph. The effect of all independent variables on the different responses of the Mentha piperita nanosuspension formulation was estimated by constructing 3D response surface plots, as stated by Hegde, et al. [37]. The collective influence of two variables was instantaneously studied in each plot; however, a third parameter was placed at its central point. The graph demonstrates that two formulation parameters (stabilizer concentration and amount of plant extract) have an important influence on particle size reduction in M. piperita nanosuspensions. However, the effect of the amount of plant extract is more noticeable, as shown in Figure 3. The lowest PDI and particle size are observed at 1 g of Mentha piperita extract. The classical crystallization theory describes the effect of concentration (plant extract) on particle size. According to this concept, the precipitation of nanoparticles during nanoformulation involves multiple steps, including nucleation, molecular growth, coagulation, condensation, and agglomeration. Moreover, the rate of each step is important for the final distribution of particle size. This mechanism is regulated by supersaturation, which controls nucleation and diffusion rates. Particle nucleation and growth occur concurrently and compete for supersaturation. Due to greater supersaturation at a high drug concentration, the agglomeration rates and diffusion-controlled growth were more prominent than the nucleation rate, which gives rise to bigger particles.

### 3.4. Biological Activities of Mentha piperita Nanosuspension

The following biological activity tests were performed for the evaluation of the antibacterial, antifungal, and pesticidal potential of *Mentha piperita*-based nanobiopesticides.

#### Pesticidal Activity

**Figure 4:**
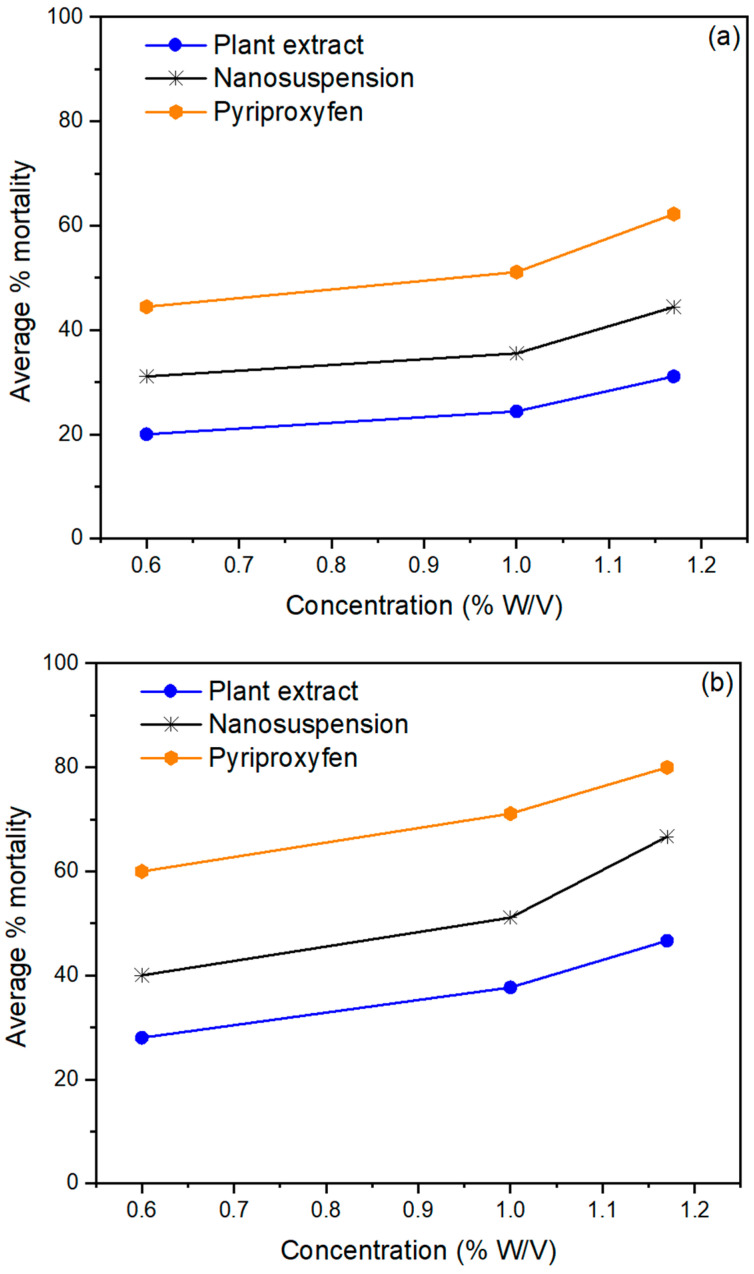

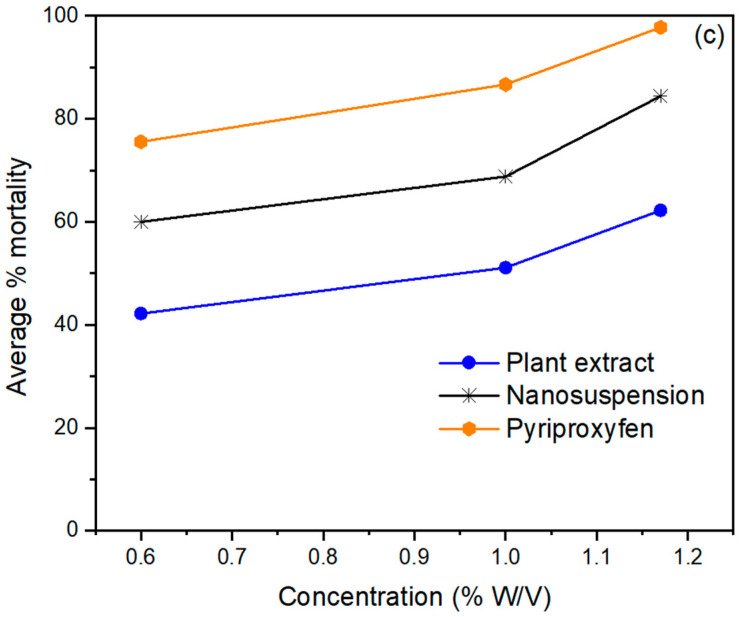
Average % mortality of *Mentha piperita* extract and nanobiopesticide *T. castaneum* at different concentrations after (**a**) 24 h, (**b**) 48 h, (**c**) 72 h.

The pesticidal potential of *Mentha piperita*-based nanobiopesticides was determined against two wheat grain pests, *Sitophilus oryzae* (Figure 4a) and *Tribolium castaneum* (Figure 4b), at different concentrations and exposure times. After 72 h of exposure, the *Mentha piperita*-based nanobiopesticides demonstrated the highest mortality rates: 84.4% and 77.77% against *Tribolium castaneum* and *Sitophilus oryzae,* respectively. The lowest mortality rates of *T. castaneum* and *S. oryzae* were 31.1% and 26.66%, respectively, which were observed at the minimum concentration of 0.60% after 24h. Similarly, the *Mentha piperita* extract exhibited the highest mortality of 62.22% and 57.7% against *T. castaneum* and *S. oryzae* at the maximum concentration of 1.17% after 72h of treatment. The lowest mortality of 20% and 17.7%, respectively, was observed at the minimum concentration of 0.60% after 24 h.

The findings demonstrated that *Mentha piperita*-based nanobiopesticides had significantly (*p* < 0.05) greater potential for suppressing both stored-grain pests than *Mentha piperita* crude extract. The most important problem for the storage of grains is the risk of pest infestation, which has a significant impact on the worldwide economy. *Sitophilus oryzae* (Rice weevils) are destructive pests of stored foods like rice, wheat, and maize. Rice weevil infestation in grains causes quality reduction, microbial growth, and weight loss by elevating the level of free unsaturated fat. *Tribolium castaneum* (red flour beetle) is a cosmopolitan, polyphagous pest in cereal products, flour mills, and stored dried foodstuffs. The infestation caused by pests is mainly controlled by using synthetic insecticides, but their uncontrolled use causes insects to develop resistance against these pesticides, which affect health along with the environment. The *M. piperita*-based nanobiopesticides have good potential as green biopesticides because of their high contents of pesticidal bioactive phytochemicals.

In a previous report, a prepared nanoemulsion of *Mentha piperita* essential oil showed relatively high contact toxicity against cotton aphids. A smaller particle size accelerates insecticide penetration through the insect cuticle and plays a crucial role in insecticidal potential [38,39]. Pyriproxyfen showed 97.77% and 93.33% mortality against *T. castaneum* and *S. oryzae* at the maximum concentration after 72 h of contact. The synthetic control Pyriproxyfen showed the highest pesticidal activity against red flour beetles and rice weevils (97% and 93%, respectively) as compared to the *Mentha piperita* nanosuspension and extract.

Under laboratory conditions, the observed mortality of the *Mentha piperita* extract and nanosuspensions was exposure-time- and concentration-dependent. Our findings align with the fact that increases in the duration of exposure and the concentration of the extract increase the mortality of *T. castaneum* and *S. oryzae*. This could be due to the increase in bioactive components as the concentration of the extract increased. The maximum mortality rates were shown at the maximum concentration (1.17%), while the minimum mortality rates were observed at the lowest concentration (0.60%). Many other oils, like fennel oil, showed 91.48% mortality against *T. castaneum* at 5% (*v*/*v*), which increased to 96.68% at a 10% (*v*/*v*) concentration. The *Mentha longifolia* extract exhibited 100% repellent potential against the lesser grain borer at a 75% concentration after 10 days of treatment. Mortality percentages were also directly proportional to the time after treatment. *Azadirachta indica* also expressed 85.33% repellency after a 24 h treatment, 86.67% after 48 h, and 93.33% after 72 h of exposure [40].

The previous work showed that the insecticidal potential of mint oil against the German cockroach and housefly is due to monoterpenoids, important essential oil components that cause neurotoxicity in pests, and as a result, the insects die. The insecticidal activities of Mentha extracts against different pests/insects also depend upon the extraction solvent and method. M. *piperita* essential oil caused 100% mortality against *T. castaneum* and *S. oryzae* at 100 μL/L and 75 μL/L, respectively, by inhibiting AChE activity. The *M. piperita* EO and extract have been reported for their insecticidal and antifeedant activities against a wide range of pests: *S. oryza, T. castaneum, Musca domestica, R. dominica,* and *Ips typographus.* The variation in the toxicity of nanosuspensions and extracts to pests is credited to the level of vulnerability; biochemical, physiological, and metabolic responses; and structural differences, e.g., texture, body size, and cuticle thickness [40,41,42,43]. Furthermore, the *Mentha piperita* nanosuspension had characteristics of high activity and low toxicity; therefore, it could be developed as an ideal pesticide for the agricultural industry.

### 3.5. Antibacterial Activity

**Figure 5:**
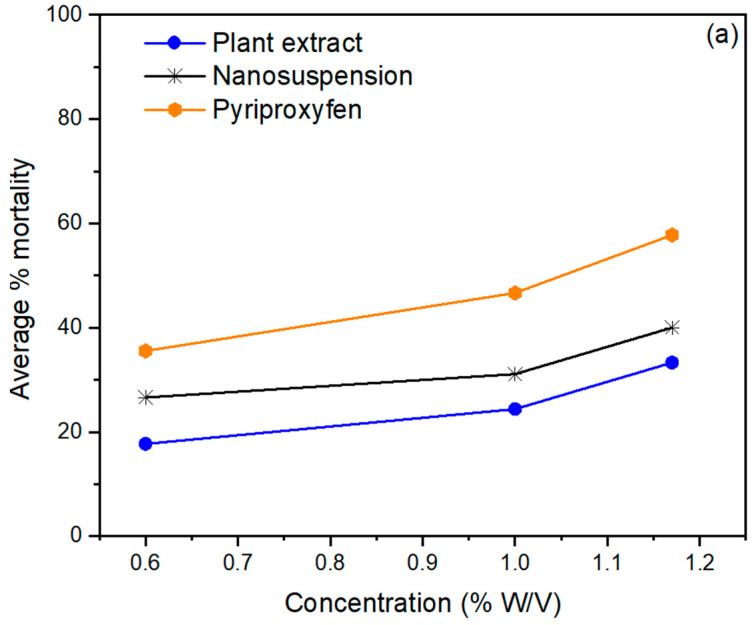

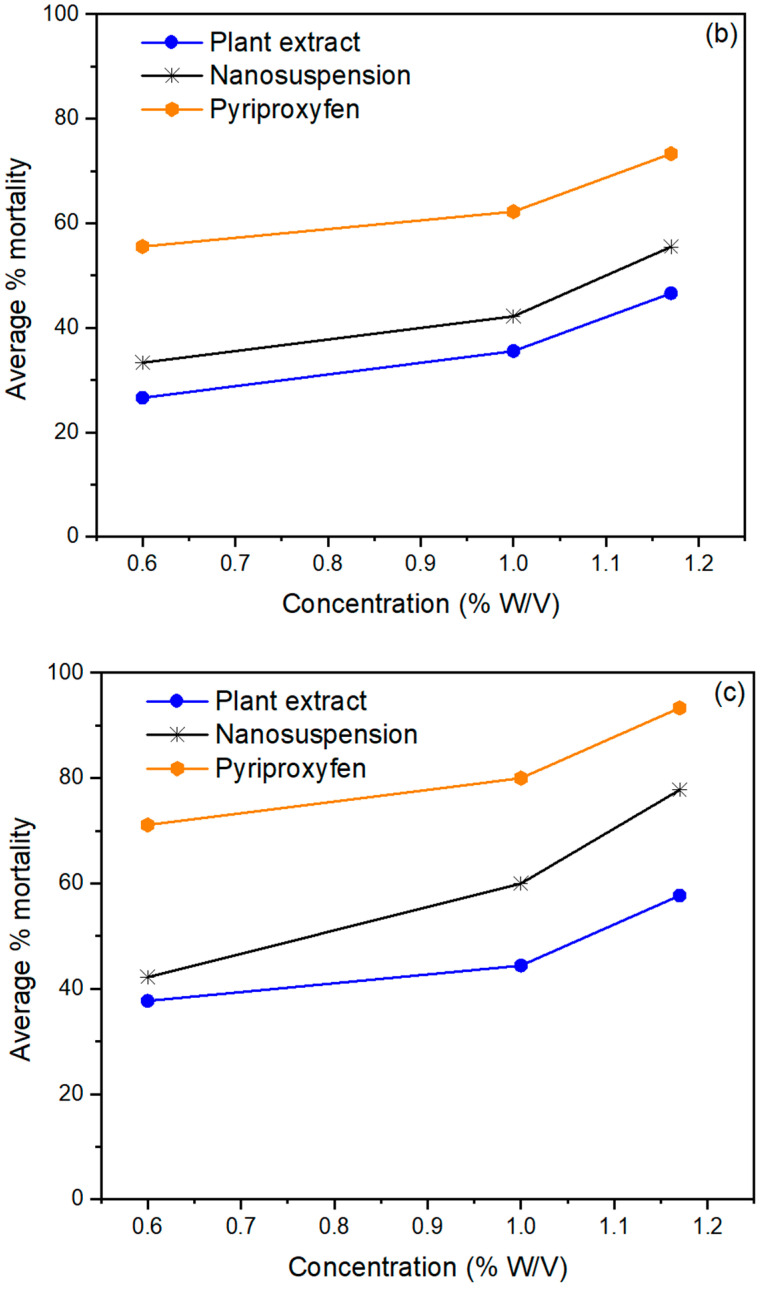
Average % mortality of *Mentha piperita* extract and nanobiopesticide against *Sitophilus oryzae* at different concentrations after (**a**) 24 h, (**b**) 48 h, (**c**) 72 h.

The antibacterial activity of the *Mentha piperita* extract and optimized nanosuspensions was investigated at three different concentrations, i.e., 0.6, 1, and 1.17 µg/mL, against Gram-positive (*Clavibacter michiganensis*) and Gram-negative bacteria (*Pseudomonas syringae*) using the agar well diffusion method as shown in Figure 5. The *Mentha piperita* nanosuspensions exhibited maximum inhibition zones against *C. michiganensis* (20.17 ± 1.07) and *P. syringae* (16.18 ± 1.06) at a 1.17% concentration. The *Mentha piperita* extract revealed maximum inhibition zones against *C. michiganensis* (16.27 ± 0.86) and *P. syringae* (13.9 ± 0.602) at a 1.17 µg/mL concentration. The results indicated that *Mentha piperita*-based nanobiopesticides showed significantly stronger (*p* < 0.05) antibacterial activity against *C. michiganensis* and *P. syringae* as compared to coarse *Mentha Piperita* suspensions at similar concentrations. The increased activity was due to the small particle size, which increased the surface area [44]. The nanosuspension interacts easily with the bacterial cell wall, rupturing it and strongly inhibiting the growth of Gram-positive bacteria [45].

It was also observed from the results that *Mentha piperita* resulted in larger inhibition zones against *C. michiganensis* as compared to *P. syringae*. The lipopolysaccharides present in the outer membrane of Gram-negative bacteria might be responsible for their enhanced resistance to antibacterial substances. *Clavibacter michiganensis* is an aerobic, non-motile, Gram-positive bacterium that causes bacterial canker disease in tomatoes all over the world. *Pseudomonas syringae* is a Gram-negative phytopathogenic bacterium that causes serious damage to fruits and vegetables. Studies from the previous literature have proven that Gram-positive bacteria are more sensitive to the *Mentha piperita* EO and extract [46,47].

Secondary metabolites, e.g., tannins, flavonoids, terpenoids, and alkaloids, are responsible for the antibacterial activities of the *Mentha piperita* extract [48]. Ciprofloxacin exhibited maximum inhibition zones against *C. michiganensis* (25.3 ± 0.33) and *P. syringae* (22.4 ± 0.24) at a 1.17% concentration. The control showed the maximum inhibition zone values against these bacterial strains as compared to the *Mentha extract* and nanosuspensions. A previous study showed that *Mentha piperita* effectively inhibited the growth of *S. aureus, E. coli, X. campestris*, and *C. tropicalis* [49]. It was also evaluated experimentally, and all the details and data are given in Table 3. In addition, the plate images from antimicrobial activity experiments are given in the Supplementary Materials in Figures S1–S3. All these dilutions showed dose-dependent activity. The maximum antibacterial potential was observed at the highest concentrations (1.1 µg/mL) of the extract, nanosuspension, and control. The increasing concentration of the extract increased the inhibition zone diameter, which is due to the increasing strength of bioactive components in the extract to destroy the bacterial population. Given the possible dangers of chemical pesticides, the *Mentha piperita*-based nanobiopesticides can be used as biopesticides to control phytopathogenic microbes, as they are safe for the environment and human health.

### 3.6. Antifungal Activity

The *Mentha piperita* nanosuspensions exhibited the maximum inhibition zones against *A. niger* (19.7 ± 1.15) and *F. oxysporum* (25.5 ± 1.35) at a 1.17 µg/mL concentration, as given in Table 4. Minimum inhibition zones of 13.3 mm and 15.4 mm were observed at the lowest concentration of 0.60 µg/mL, respectively. Similarly, the *Mentha piperita* extract revealed maximum inhibition zones against *F. oxysporum* (20.3 ± 1.06) and *A. niger* (15.5 ± 0.71) at a 1.17 µg/mL concentration. The *Mentha piperita* nanosuspension showed stronger antifungal activity against *F. oxysporum* and *A. niger* as compared to coarse *Mentha Piperita* suspensions. The high antifungal activity of the nanosuspension can be attributed to the larger surface area, resulting in higher/increased contact with the target sites on cell membranes. In addition, nanoparticles with smaller particle sizes can easily penetrate through the plasma membrane. Similarly, *Mentha piperita*-based AgNPs exhibited antifungal activity against *C. albicans* [50,51]. All the data regarding zones of inhibition for *F. oxysporum* and *A. niger* are given in Table 4. In addition, the plate images from antifungal activity experiments are given in the Supplementary Materials in Figures S4–S7.

The antifungal activity of *M. piperita* oil can be mainly attributed to its high content of oxygenated monoterpenoids, such as menthone and menthol. It was revealed from TEM and SEM analysis that mint oil exerted pesticidal activity by damaging the structures of the conidia and hyphae. Ref. [52] reported that the menthol present in MPEO causes the disruption of the cell membrane, an increase in membrane permeability, and the subsequent leakage of cytoplasmic content. The *Mentha extract* and its EO also showed antifungal activities against *Pseudomonas solanacerum*, *A. niger*, *Penicillium funiculosum*, *Trichoderma viride*, *Rhizopus solani*, *Cryptococcus neoformans*, *C. albicans Alternaria alternata*, and *Fusarium chlamydosporum* [52]. The control (voriconazole) showed maximum inhibition zones against *F. oxysporum* (35.4 ± 0.39) and *A. niger* (30.3 ± 0.2) at a 1.17 µg/mL concentration. It was also determined from the graphs that all these dilutions showed dose-dependent activity [52]. The maximum antifungal potential was observed at the highest concentration (1.17%) of the extract, nanosuspension, and control, while the minimum antifungal activity was observed at the lowest (0.60 µg/mL) concentration. The antifungal activity of CuNPs against *F. oxysporum* and *P. capsici* increased when increasing the concentration from 5 ppm to 20 ppm, even at different particle sizes.

Differences in the sensitivity of *F. oxysporum* and *A. niger* might be because of the presence of a complex rigid cell wall or their resistance to toxic chemicals [53]. *Aspergillus niger* causes a disease called black mold on fruits and vegetables like grapes, onions, and peanuts and is a common contaminant of food. Vascular wilt caused by *Fusarium oxysporum* is also a serious soil-borne disease of tomato. The use of conventional fungicides to control the disease causes several injurious effects. Therefore, the *Mentha piperita* nanosuspension can play an important role in causing physical damage to fungi, facilitating its use as an effective antifungal agent.

## 4. Conclusions

A *Mentha piperita* nanobiopesticide with a suitable particle size was successfully formulated for the first time at optimized conditions of a 1% *Mentha piperita* extract concentration and 0.2% SLS concentration. These nanobiopesticides showed enhanced pesticidal, antibacterial, and antifungal potential as compared to the crude extract. The nanosizing of the formulation improved its contact with the insects, which significantly increased the effectiveness of the *Mentha piperita*-based nanobiopesticide when compared to the traditional extract. Overall, this research indicates that the formulated nanoformulations are effective pesticides with potential for commercialization in the agrochemical industries.

## Figures and Tables

**Table 1:** Experimental design suggested by central composite design (CCD) of response surface methodology (RSM) for optimization of important parameters.

Sr No.	Amount of Plant Extract (g)	Concentration of Stabilizer (%)	Particle Size (nm)	Polydispersity Index (PDI)
1	0.60	0.78	309.2	0.378
2	1.17	0.78	285.2	0.493
3	0.60	0.78	261.2	0.478
4	0.60	0.78	302.2	0.548
5	1	1.35	414.9	0.614
6	0.20	0.20	431	0.637
7	0.60	−0.04	305	0.43
8	0.60	0.78	260.2	0.398
9	0.03	0.78	399.4	0.534
10	1	0.20	259.8	0.626
11	0.60	1.59	282.9	0.608
12	0.20	1.35	419.7	0.512
13	0.60	0.78	261.5	0.518

**Table 2:** Analysis of variance (ANOVA) for particle size of nanosuspension.

Source	Sum of Squares	df	Mean Square	F-Value	*p*-Value Probe > F	
Model	40,539.13	5	8107.83	3.97	0.0499	Significant
A—Amount of plant extract	14,238.55	1	14,238.55	6.98	0.0333	
B—Stabilizer concentration	1583.32	1	1583.32	0.78	0.4075	
AB	6922.24	1	6922.24	3.39	0.1080	
A^2^	15,713	1	15,713.00	7.70	0.0275	
B^2^	3793.26	1	3793.26	1.86	0.2149	
Residual	14,278.28	7	2039.75			
Lack of fit	11,851.56	3	3950.52	6.51	0.0510	Non-significant
Pure error	2426.71	4	606.68			
Total	54,817.4	12				

**Table 3:** Zones of inhibition for *C. michiganesis* and *P. syringae*.

Antibacterial Activity	Concentration µg/mL	Zones of Inhibition (mm)	
		*P. syringae*	*C. michiganesis*
*M. piperita* extract	0.60	10.1 ± 0.95 ^Cc^	12 ± 1.45 ^Cc^
	1	12.06 ± 0.95 ^Bc^	14.41 ± 0.28 ^Bc^
	1.17	13.9 ± 0.602 ^Ac^	16.27 ± 0.86 ^Ac^
Nanosuspension	0.60	12.1 ± 0.88 ^Cb^	14.33 ± 0.83 ^Cb^
	1	13.76 ± 1.1 ^Bb^	18.2 ± 0.9 ^Bb^
	1.17	16.18 ± 1.06 ^Ab^	20.17 ± 1.07 ^Ab^
Ciprofloxacin (C)	0.60	15.3 ± 0.26 ^Ca^	20.2 ± 0.2 ^Ca^
	1	17.01 ± 0.46 ^Ba^	22.37 ± 0.29 ^Ba^
	1.17	22.4 ± 0.24 ^Aa^	25.3 ± 0.33 ^Aa^

The capital letters indicate that the maximum results were obtained at the highest concentration, while lowercase letters show that the maximum activity was achieved by the control, nanosuspension, and plant extract, respectively.

**Table 4:** Zones of inhibition for *F. oxysporum* and *A. niger*.

Antifungal Activity	Concentration µg/mL	Zones of Inhibition (mm)	
		*F. oxysporum*	*A. niger*
*M. piperita* extract	0.60	12.4 ± 1.96 ^Cc^	11.24 ± 0.77 ^Cc^
	1	16.26 ± 0.86 ^Bc^	12.86 ± 0.99 ^Bc^
	1.17	20.3 ± 1.06 ^Ac^	15.5 ± 0.71 ^Ac^
Nanosuspension	0.60	15.4 ± 1.25 ^Cb^	13.3 ± 1.05 ^Cb^
	1	20.5 ± 2.25 ^Bb^	16.4 ± 0.75 ^Bb^
	1.17	25.5 ± 1.35 ^Ab^	19.7 ± 1.15 ^Ab^
Voriconazole(C)	0.6	20.24 ± 0.22 ^Ca^	16.2 ± 0.17 ^Ca^
	1	30.4 ± 0.27 ^Ba^	25.23 ± 0.86 ^Ba^
	1.17	35.4 ± 0.39 ^Aa^	30.3 ± 0.2 ^Aa^

The capital letters indicate that the maximum results were obtained at the highest concentration, while lowercase letters show that the maximum activity was achieved by the control, nanosuspension, and plant extract, respectively.

## Data Availability

Data are contained within the article and Supplementary Materials.

## Supplementary Materials

The following supporting information can be downloaded at https://www.mdpi.com/article/10.3390/life14010144/s1, Figure S1. Zones of inhibition against *C. michiganesis* and *P. syringae* with the control ciprofloxacin. Figure S2: Zones of inhibition for Mentha piperita extract and nanosuspensions against *C. michiganensis*. Figure S3: Zones of inhibition for Mentha piperita extract and nanosuspensions against *P. syringae*. Figure S4: Zones of inhibition for Mentha piperita extract and nanosuspensions against *F. oxysporum*. Figure S5. Zones of inhibition for Mentha piperita extract and nanosuspensions against *A. niger*. Figure S6: Zones of inhibition against *F. oxysporum* with the control voriconazole. Figure S7: Zones of inhibition against *A. niger* with the control voriconazole.
