# Supplementary material for: Formulation of Mentha piperita-Based Nanobiopesticides and Assessment of the Pesticidal and Antimicrobial Potential
Source: Life (Basel). 2024 Jan 19;14(1):144. doi: 10.3390/life14010144 (PMC10817515; doi:10.3390/life14010144)
Supplement: Supplementary file 1 [file life-14-00144-s001.zip › life-2580271-supplementary.pdf]

life

Supplementary information

Formulation of Mentha piperita -Based Nano Biopesticides and Assessment of the Pesticidal and Antimicrobial Potential

Nazish Jahan [1,†], Nida Hussain [1], Syeeda Iram Touqeer [1], Khalil-Ur-Rahman [2], Huma Shamshad [1] and Naseem Abbas [3,*,†]

[1] Department of Chemistry, University of Agriculture, Faisalabad 38000, Pakistan; nazishjahan@uaf.edu.pk (N.J.); nidahussain116@gmail.com (N.H.); iram_touqeer@yahoo.com (S.I.T.); dogarhuma@gmail.com (H.S.)
[2] Department of Biochemistry, Riphah International University, Faisalabad 38000, Pakistan; khaliluaf@yahoo.com
[3] Department of Mechanical Engineering, Sejong University, Seoul 05006, Republic of Korea
* Correspondence: naseem.abbas@sejong.ac.kr
† These authors contributed equally to this work.

The plate images for antimicrobial activity experiments are given in supplementary materials with Figure S1-to Figure S3 whereas, the plate images for antifungal activity experiments are given in supplementary materials with Figure S4-to Figure S7.

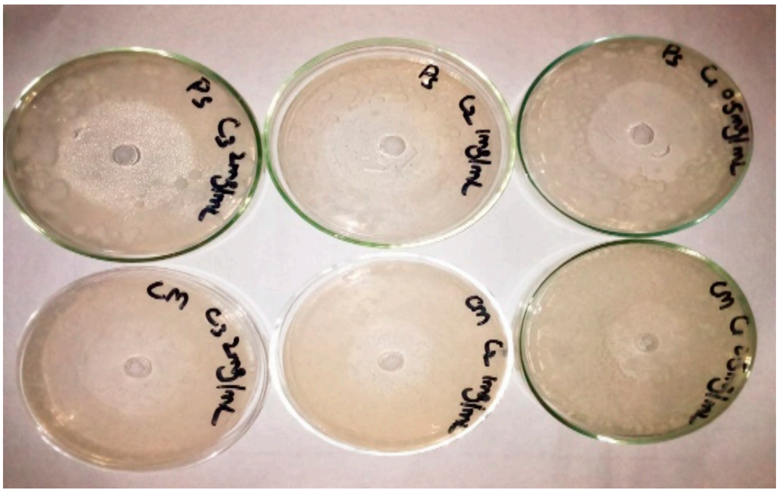

Figure S1. Zones of inhibition against *C. michiganesis* and *P. syringae* for control Ciprofloxacin.

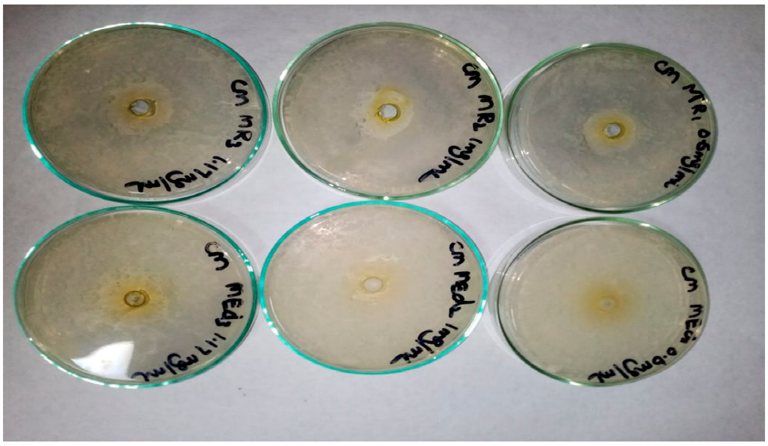

Figure S2. Zones of inhibition for *Mentha piperita* extract and nanosuspensions against *C. michiganensis.*

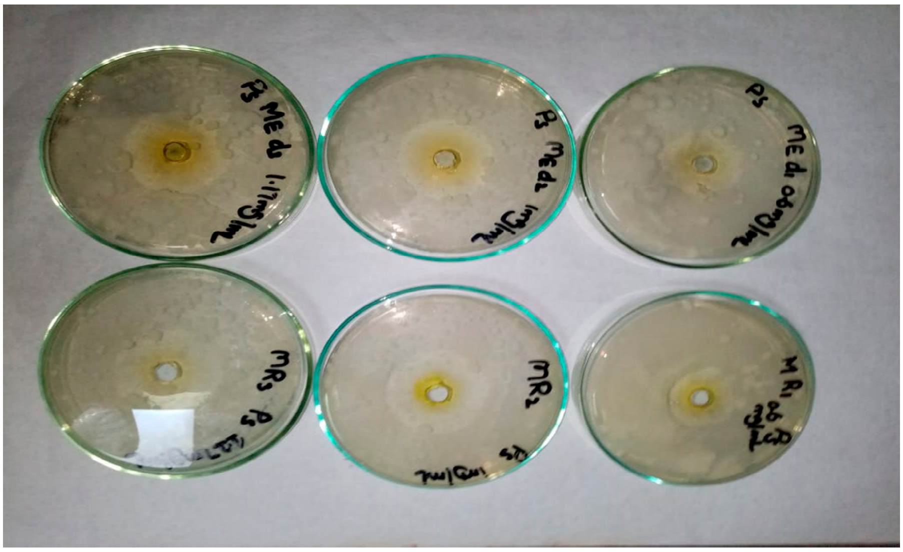

Figure S3: Zones of inhibition for *Mentha piperita* extract and nanosuspensions

against *P. syringae*

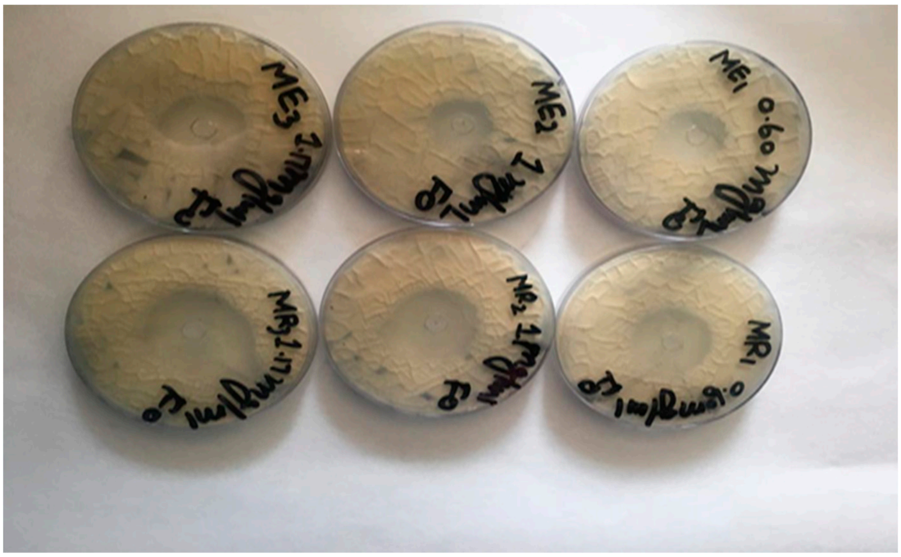

Figure S4: Zones of inhibition for *Mentha piperita* extract and nanosuspensions against *F. oxysporum*

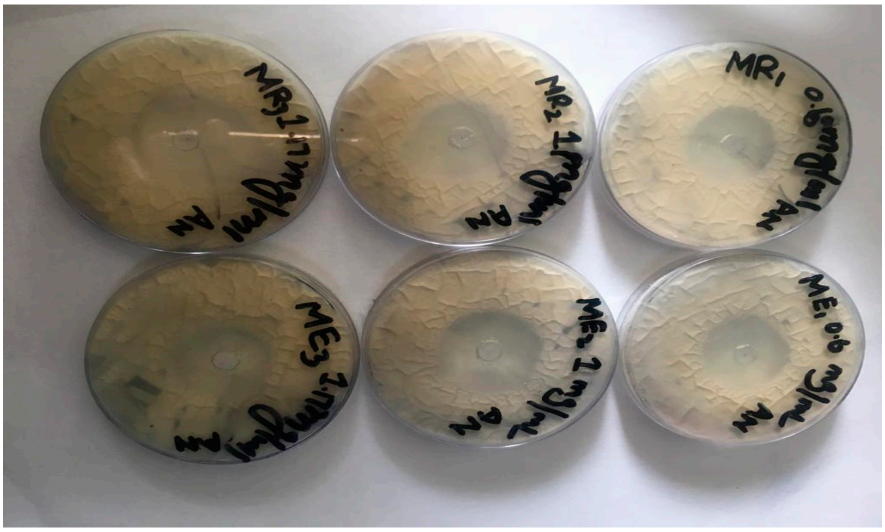

Figure S5. Zones of inhibition for *Mentha piperita* extract and nanosuspensions against *A. niger*

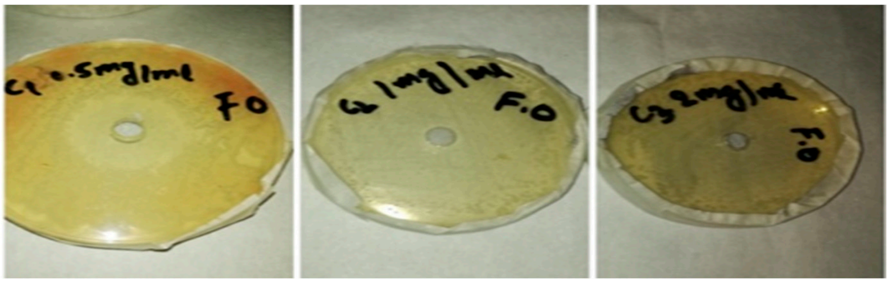

Figure S6: Zones of inhibition against *F. oxysporum* for control Voriconazole

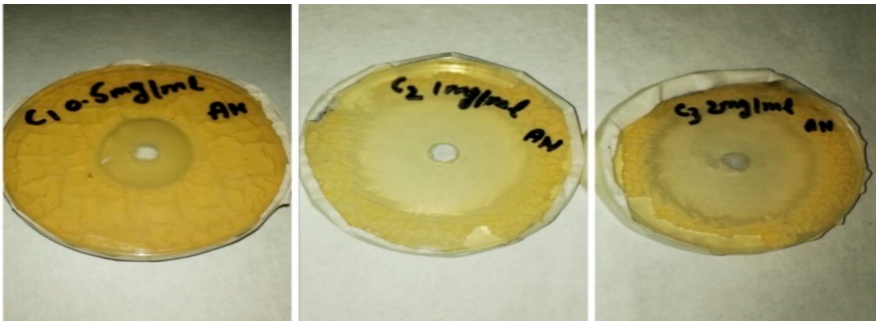

Figure S7: Zones of inhibition against *A. niger* for control Voriconazole
